# Psychometric properties of the Japanese version of the standardised assessment of personality abbreviated scale

**DOI:** 10.3389/fpsyg.2023.1339902

**Published:** 2024-02-06

**Authors:** Issaku Kawashima, Tomoko Hinuma, Masatoshi Nagata, Akio Yoneyama, Masaru Honjo, Hiroaki Kumano, Saori C. Tanaka

**Affiliations:** ^1^Brain Information Communication Research Laboratory Group, Advanced Telecommunications Research Institute International (ATR), Kyoto, Japan; ^2^Healthcare Medical Group, Life Science Laboratories, KDDI Research, Inc., Tokyo, Japan; ^3^Life Science Laboratories, KDDI Research, Inc., Tokyo, Japan; ^4^Faculty of Human Sciences, Waseda University, Saitama, Japan; ^5^Division of Information Science, Graduate School of Science and Technology, Nara Institute of Science and Technology (NAIST), Nara, Japan

**Keywords:** personality disorder, analog study, questionnaire, assessment tool, validity and reliability, standardised assessment of personality abbreviated scale

## Abstract

This study was undertaken to translate the Standardised Assessment of Personality – Abbreviated Scale (SAPAS) into Japanese and to evaluate its validity and reliability. SAPAS is one of the most rapid tools for assessing personality disorder (PD) and has excellent sensitivity and good specificity, whereas other PD assessment tools require such a significant investment of time that they are infeasible for large surveys or routine clinical practice. Customary assessment in clinical practice ideally incorporates screening for PD, as it is associated with a substantial public health burden, including premature mortality and increased health service utilization. Furthermore, PD’s status as a key prognostic variable of mental disorders also drives PD screening. While SAPAS has been translated into several languages, there has been no Japanese version. Therefore, we translated SAPAS into Japanese (SAPAS-J) and evaluated its reliability and validity. Study 1 recruited undergraduates to reveal its test–retest reliability. Although its internal consistency was not high, since the intent of the original SAPAS was to assess the broad character of personality disorder with the fewest possible items, minimal correlations between items were reasonable. We tested two factorial models, the single-factor model and the higher-order-single-factor model, and the latter offered better fitting. This higher-order model contained a three-factor structure corresponding to clusters described in DSM-5. It measures general PD traits as a common higher-order latent variable comprising those factors. Correlations of SAPAS-J with the much longer PD screening questionnaire in Study 1 and depressive and anxiety symptoms in Study 2 from the general population support its validity. Although validation for the clinical use of SAPAS-J is limited, our research with non-clinical populations demonstrated sufficient validity to justify its use in the context of psychopathological analog research. Since PD is understood as a continuum, the severity of which is distributed dimensionally, the analog study recruiting from the general population and attempting to reveal psychopathological mechanisms of PD is meaningful.

## 1 Introduction

The purpose of this study was to translate the Standardised Assessment of Personality – Abbreviated Scale (SAPAS; Moran et al., 2003) into Japanese and to evaluate its validity and reliability. SAPAS is one of the most efficient screening tools for personality disorder (PD), the mental disorder characterized by pervasive, inflexible, and enduring experiential/behavioral patterns that deviate from the expectations of the individual’s culture and cause distress and impairment of functioning (American Psychiatric Association, 2013b). SAPAS has excellent sensitivity and good specificity to screen patients with PD (Moran et al., 2003) and captures variance specifically associated with PD rather than variance of general personality traits (Ball et al., 2017). The SAPAS score also correlates with several PD-related symptoms, including depression, anxiety, and impairment of social function (Goddard et al., 2015). While it was originally developed as a structured interview, it was also validated as a questionnaire (Germans et al., 2008) in clinical populations. Moreover, SAPAS is one of the most rapid tools for assessing PD since it has only eight items, whereas other PD assessment tools, including structured interviews (1–2 h to complete) and questionnaires (85–390 items) are infeasible for routine clinical practice or large surveys (Tyrer et al., 2015).

Routine assessment in clinical practice ideally incorporates screening of PD (Moran et al., 2003; Fok et al., 2015), given that PD constitutes a major mental health burden. Life expectancy at birth among patients with PD is 18.7 years less for women and 17.7 years less for men than for the general population (Fok et al., 2012). Reported quality-of-life scores of patients with PD are lower than those of patients with major depressive disorder (Laurenssen et al., 2016). The economic cost of PD was estimated as €16,879 per year (Laurenssen et al., 2016) and it is reported that PD increases health service costs not only in psychiatry but also in non-mental health services (Kavanagh et al., 2021). One of the reasons for this is treatment-seeking motivated by difficulties in accessing appropriate care (Kavanagh et al., 2021). Routine PD screening in non-mental health services would help to overcome this problem.

Furthermore, the fact that PD is a key prognostic variable of mental disorders motivates PD screening. The comorbidity of PD decreases the remission rate of main symptoms in several mental disorders, including anxiety disorders (Massion et al., 2002; Carlier et al., 2014), body dysmorphic disorder (Phillips et al., 2005), depression, and somatoform disorder (Carlier et al., 2014). While the global prevalence of PD is estimated at 7.8% (Winsper et al., 2020), the comorbidity rate among mental disorders is much higher. For example, 45–77% of patients with major depressive disorder exhibit PD comorbidity (Friborg et al., 2014; Wongpakaran et al., 2015). Hence, screening tools for PD help formulate prognoses for treatment. The SAPAS score predicts the dropout rate (Goddard et al., 2015) and suboptimal responses from psychotherapy (Goddard et al., 2015; Mars et al., 2021) and antidepressants (Gorwood et al., 2010). Furthermore, SAPAS items contribute to personalized psychotherapy recommendations based on machine learning (Delgadillo et al., 2017, 2022).

In brief, SAPAS is the most efficient screening tool for PD. It helps to introduce patients to appropriate treatment and predicts prognoses of comorbid mental disorders. While SAPAS has now been translated into several languages, including Spanish, French, Danish, and Bengali (Sen et al., 2021), there has been no Japanese version. Therefore, we translated SAPAS into Japanese (SAPAS-J) and evaluated its reliability and validity.

## 2 Materials and methods

To establish the validity and reliability of the SAPAS, we performed two studies. The first study investigated internal consistency, test–retest reliability, and convergent and structural validity. The second study compensated for some limitations of Study 1 and supported its convergent validity.

Study protocols were approved by an ethics committee and were not preregistered. To perform these analyses, we used Python (version 3.10.6), R (version 4.2.2), Pingouin-stats (version 0.5.3; Vallat, 2018), and Lavaan (version 0.6.13; Rosseel, 2012).

### 2.1 Study 1

#### 2.1.1 Participants

In the first study, 110 (31 men) undergraduates at a university in Japan provided valid answers. The average age was 23.27 years (SD = 6.77). Among participants, 58 responded to the Depression and Anxiety Mood Scale (DAMS; Fukui, 1997) and 97 to the Personality Diagnostic Questionnaire-Revised (PDQ-R; Hyler et al., 1992). Two weeks after the first survey, we retested using SAPAS-J, and 33 participants responded.

#### 2.1.2 Measures

##### 2.1.2.1 SAPAS-J

SAPAS-J, the questionnaire translated in the current study, is an eight-item assessment of PD tendency. The authors, including a medical doctor of psychosomatic medicine, translated the original SAPAS into Japanese. While the original SAPAS and the self-reporting version of SAPAS (Germans et al., 2008) used dichotomous (yes/no) questions, SAPAS-J employs a 4-response Likert-scale (1. False for me; 2. Somewhat false for me; 3. Somewhat true for me; 4. True for me). Binary questions can be completed more quickly; however, nuanced answers are forced into “yes” or “no” and tend to be “yes” (Rivera-Garrido et al., 2022). Therefore, we adopted a scaled rating in order to capture more subtle psychopathology to improve its specificity. The author of the original SAPAS checked the back-translated SAPAS-J and confirmed that the meanings of the original questions were retained. SAPAS-J is available in the Supplementary material and is available for anyone to use for research purposes.

##### 2.1.2.2 Personality diagnostic questionnaire-revised

PDQ-R is a self-report assessment tool for PD. The original (Hyler et al., 1992) and the Japanese version (Uehara et al., 1997) of PDQ-R show sufficient validity and reliability. Diagnostic comparisons with semi-structured interview assessments revealed that PDQ-R has low specificity, but fairly high sensitivity (Hyler et al., 1992). However, we used this questionnaire to validate SAPAS because a high-sensitivity/low-specificity assessment tool may produce sufficient variation among non-clinical populations. PDQ-R was also employed among non-clinical populations to assess general PD traits using its total score (Trull et al., 1993), although it was originally developed to detect patients with PD. We abbreviated the 31 duplicated items and items in a dummy factor and used 121 items.

##### 2.1.2.3 Depression and anxiety mood scale

DAMS, a 7-point Likert 9-item questionnaire having high validity and reliability, assesses depressive and anxious moods in the preceding two or 3 days (Fukui, 1997; Takahashi et al., 2021). DAMS has three factors: positive mood, negative mood, and anxious mood.

#### 2.1.3 Analyses

We performed two patterns of confirmatory factorial analyses. As in the original SAPAS, we assumed that the SAPAS-J total score measures transdiagnostic general PD traits, and prepared a single-factor model in which all items were explained by one factor. Additionally, we tested the higher-order model in which items were classified into three factors, corresponding to clusters described in the Diagnostic and Statistical Manual of Mental Disorders 5 (DSM-5; American Psychiatric Association, 2013b). Since PD includes a wide range of symptoms and SAPAS was developed based on the cluster model of DSM, we considered that the three-factor model would explain the structure of SAPAS better than a single-factor model. However, SAPAS was originally developed as a scale for measuring only one construct, i.e., general PD trait. Furthermore, considering the brevity of SAPAS, the interpretation of each subscale is not informative. Hence, in the model, we devised one higher-order factor that included those three factors. We calculated *CFI*, *RMSEA*, and *SRMR* as indices of goodness-of-fit. Considering the model’s simplicity and the relatively small dataset (< 500), we defined acceptable cutoffs as *CFI* > 0.90, *RMSEA* < 0.10, and *SRMR* < 0.10 (Weston and Gore, 2006).

To investigate the reliability of SAPAS-J, we calculated Cronbach’s alpha *α_Cronbach_*, McDonald’s omega *ω_McDonald_*, and interclass correlation (*ICC*) between the test and retest scores. To validate SAPAS-J, we investigated correlations between the SAPAS-J score and the subscale and total score of PDQ-R and DAMS. *p* values of correlations were adjusted with Holm’s method.

### 2.2 Study 2

We collected answers from 2,004 people (1,000 men), and their average age was 49.3 years (SD = 17.0). We calculated correlations between SAPAS-J scores and tendencies toward symptoms of depression, general anxiety, social anxiety, and obsessive compulsiveness. These symptoms frequently co-occur with personality disorder, and we hypothesized that they also correlate with the SAPAS-J score. *p* values of correlations were adjusted using Holm’s method.

#### 2.2.1 Measures

##### 2.2.1.1 SAPAS-J

##### 2.2.1.2 Center for epidemiologic studies depression scale

The CES-D is a 20-item scale assessing the depressive symptom level in the general population (Radloff, 1977). Its adequate reliability and validity were maintained in the Japanese version (Shima et al., 1985). A meta-analysis reported the comorbid rate of depressive disorder in PD as 44% (Friborg et al., 2014).

##### 2.2.1.3 State–trait anxiety inventory form Y

The STAI-Y is a widely used tool for distinguishing and assessing state and trait anxiety (Spielberger, 1983). The reliability of the Japanese version was investigated (Iwata et al., 1998). From this questionnaire, we only used the second part (STAI-Y2) to measure trait anxiety.

##### 2.2.1.4 Leibowitz social anxiety scale

The original LSAS (Liebowitz, 1987) is a 24-item clinical rating scale for social anxiety disorder (SAD) that has good reliability and validity (Heimberg et al., 1999). As its reliability and validity were also demonstrated in a self-rating Japanese version (Asakura et al., 2002), we used the self-rating LSAS. A meta-analysis summarizing comorbidities of anxiety disorders reported that 48% of patients with social anxiety disorder exhibit some cluster of PD (Friborg et al., 2013).

##### 2.2.1.5 Obsessive-compulsive inventory

The OCI is a 42-item assessment tool for the diagnosis and severity assessment of obsessive-compulsive disorder (OCD) with satisfactory reliability and validity (Foa et al., 1998). Its Japanese version also reported satisfactory reliability and validity among both undergraduate students and clinical participants (Ishikawa et al., 2014). A study reported that 52% of patients with OCD had comorbid PD (Friborg et al., 2013).

## 3 Results

### 3.1 Study 1

**Figure 1:**
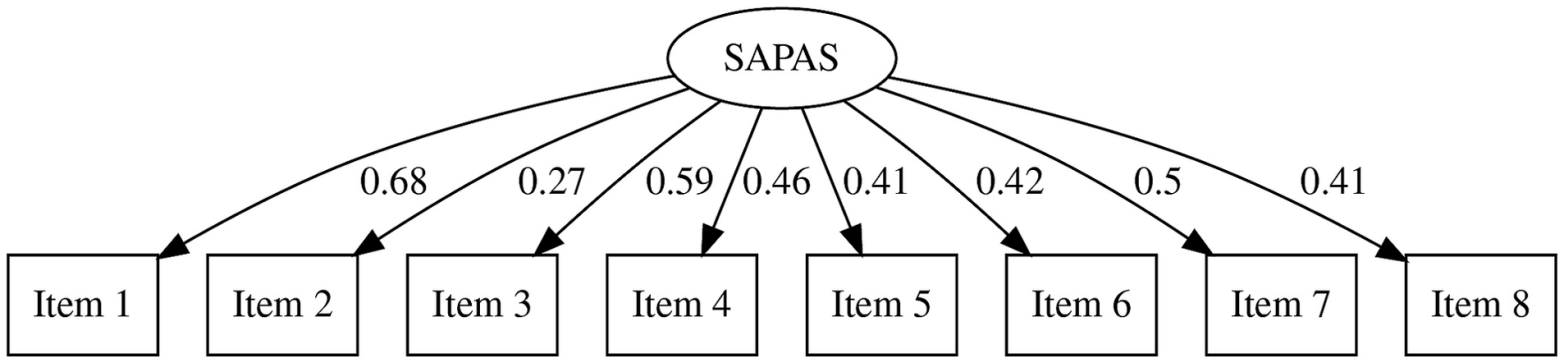
Results of confirmatory factor analysis (first-order model) *SAPAS*, the Japanese version of Standardised Assessment of Personality - Abbreviated Scale.

**Figure 2:**
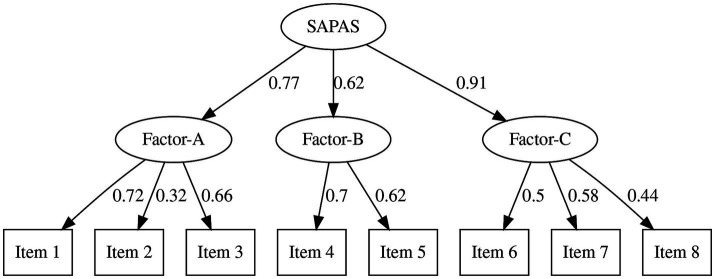
Results of confirmatory factor analysis (higher-order model) *SAPAS*, the Japanese version of Standardised Assessment of Personality - Abbreviated Scale.

Descriptive values and results of the Shapiro–Wilk test are provided in Table 1. The average score of SAPAS in Study 1 was 20.47 (SD = 4.03). There were no ceiling or floor effects. While the result of the Shapiro–Wilk test of SAPAS was not significant (*W* = 0.99, *p* = 0.274), other scores yielded a significant result (PDQ-R Cluster-A: *W* = 0.96; PDQ-R Cluster-B: *W* = 0.96; PDQ-R Cluster-C: *W* = 0.97; PDQ-R Total: *W* = 0.97; DAMS Depressive: *W* = 0.96; DAMS Anxious: *W* = 0.93; DAMS Positive: *W* = 0.96.). To investigate structural validity, we performed the CFA, assuming one-factor and higher-order structures (Figures 1, 2 and Tables 2, 3). Goodness-of-fit scores are reported in Table 4. The *CFI* of the first-order model was 0.80, *RMSEA* was 0.10, and *SRMR* was 0.08. The *CFI* of the higher-order model was 0.94. *RMSEA* was 0.06, and *SRMR* was 0.06.

**Table 1:** Descriptive statistics from results of Shapiro–Wilk tests of Study 1.

	*M*	*SE*	*W_Shapiro–Wilk_*	*p_Shapiro–Wilk_*
SAPAS				
Item 1	2.65	0.93		
Item 2	3.17	0.75		
Item 3	2.24	0.85		
Item 4	2.14	0.90		
Item 5	2.26	0.95		
Item 6	2.94	0.89		
Item 7	2.30	0.99		
Item 8	2.78	0.85		
Total score	20.47	4.03	0.99	0.274
PDQ-R				
Cluster A	6.94	3.65	0.96	0.003
Cluster B	12.90	6.67	0.96	0.004
Cluster C	11.80	4.35	0.97	0.026
Total score	35.91	14.35	0.97	0.024
DAMS				
Depressive	9.97	4.58	0.96	0.046
Anxious	12.84	5.45	0.93	0.003
Positive	12.98	4.17	0.96	0.061

Means, SDs, and results of the Shapiro–Wilk test of scores in Study 1. *W_Shapiro–Wilk_, W* values of the Shapiro–Wilk test. *p_Shapiro-Wilk_, p* values of the Shapiro-Wilk test. SAPAS, the Japanese version of Standardised Assessment of Personality - Abbreviated Scale; PDQ-R, Personality Diagnostic Questionnaire-Revised; DAMS, Depression and Anxiety Mood Scale.

**Table 2:** Factor loadings of the one-order CFA.

	Loading	*SE*	Loading (std.)
SAPAS			
Item 1			0.68
Item 2	0.32	0.14	0.27
Item 3	0.79	0.17	0.59
Item 4	0.65	0.17	0.46
Item 5	0.62	0.18	0.41
Item 6	0.59	0.17	0.42
Item 7	0.78	0.19	0.50
Item 8	0.55	0.16	0.41

Factor loadings of the one-order confirmatory factor analysis of SAPAS. Loading, Factor loading. Loading (std.), Standardised factor loading.

**Table 3:** Factor loadings of the higher-order CFA.

	Loading	*SE*	Loading (std.)
Factor-A			
Item 1			0.72
Item 2	0.36	0.13	0.32
Item 3	0.83	0.19	0.66
Factor-B			
Item 4			0.70
Item 5	0.93	0.30	0.62
Factor-C			
Item 6			0.50
Item 7	1.29	0.39	0.58
Item 8	0.84	0.29	0.44
SAPAS			
Factor-A			0.77
Factor-B	0.75	0.27	0.62
Factor-C	0.78	0.31	0.91

Factor loadings of the higher-order confirmatory factor analysis of SAPAS. Loading, Factor loading. Loading (std.), Standardised factor loading.

**Table 4:** Model fitting of CFA.

	*DF*	*χ^2^*	*p_χ2 test_*	*CFI*	*RMSEA*	*SRMR*
First-order model	20	42.27	0.003	0.80	0.10	0.08
Higher-order model	17	23.78	0.126	0.94	0.06	0.06

CFI, Comparative Fit Index; RMSEA, Root Mean Square Error of Approximation; SRMR, Standardised Root Mean square Residual.

As indices of internal consistency, we reported *α_Cronbach_* and *ω_McDonald_* (Table 5) to indicate reliability. The overall *α_Cronbach_* was 0.69 and *ω_McDonald_* was 0.70. We checked test–retest reliability using recollected answers, and the result was reliable (*ICC* = 0.85, *F* = 12.63, *p* < 0.001).

**Table 5:** Indices of reliability.

	*α_Cronbach_*	*ω_McDonald_*
First-order model	0.69	0.70
Higher-order total	0.69	0.76
Factor-A	0.60	0.61
Factor-B	0.61	0.61
Factor-C	0.51	0.52

*α_Cronbach_, Cronbach’s α. Ω_McDonald_, McDonald’s Ω.*

In order to establish the convergent validity, we calculated Spearman’s correlations (Table 6) since the Shapiro–Wilk test indicated that scores were not normally distributed. All correlations were significant and moderate (PDQ-R Cluster-A: *ρ* = 0.60; PDQ-R Cluster-B: *ρ* = 0.69; PDQ-R Cluster-C: *ρ* = 0.58; PDQ-R Total: *ρ* = 0.73; DAMS Depressive: *ρ* = 0.60; DAMS Anxious: *ρ* = 0.46; DAMS Positive: *ρ* = −0.40.)

**Table 6:** Correlations with SAPAS-J in Study 1.

	*ρ_Spearman_*	*p*	*N*
PDQ-R			
Cluster A	0.60	< 0.001	105
Cluster B	0.69	< 0.001	101
Cluster C	0.58	< 0.001	106
Total score	0.73	< 0.001	97
DAMS			
Depressive	0.60	< 0.001	58
Anxious	0.46	< 0.001	58
Positive	−0.40	0.002	58

PDQ-R, Personality Diagnostic Questionnaire-Revised. DAMS, Depression and Anxiety Mood Scale; *ρ, Spearman’s ρ*.

### 3.2 Study 2

**Figure 3:**
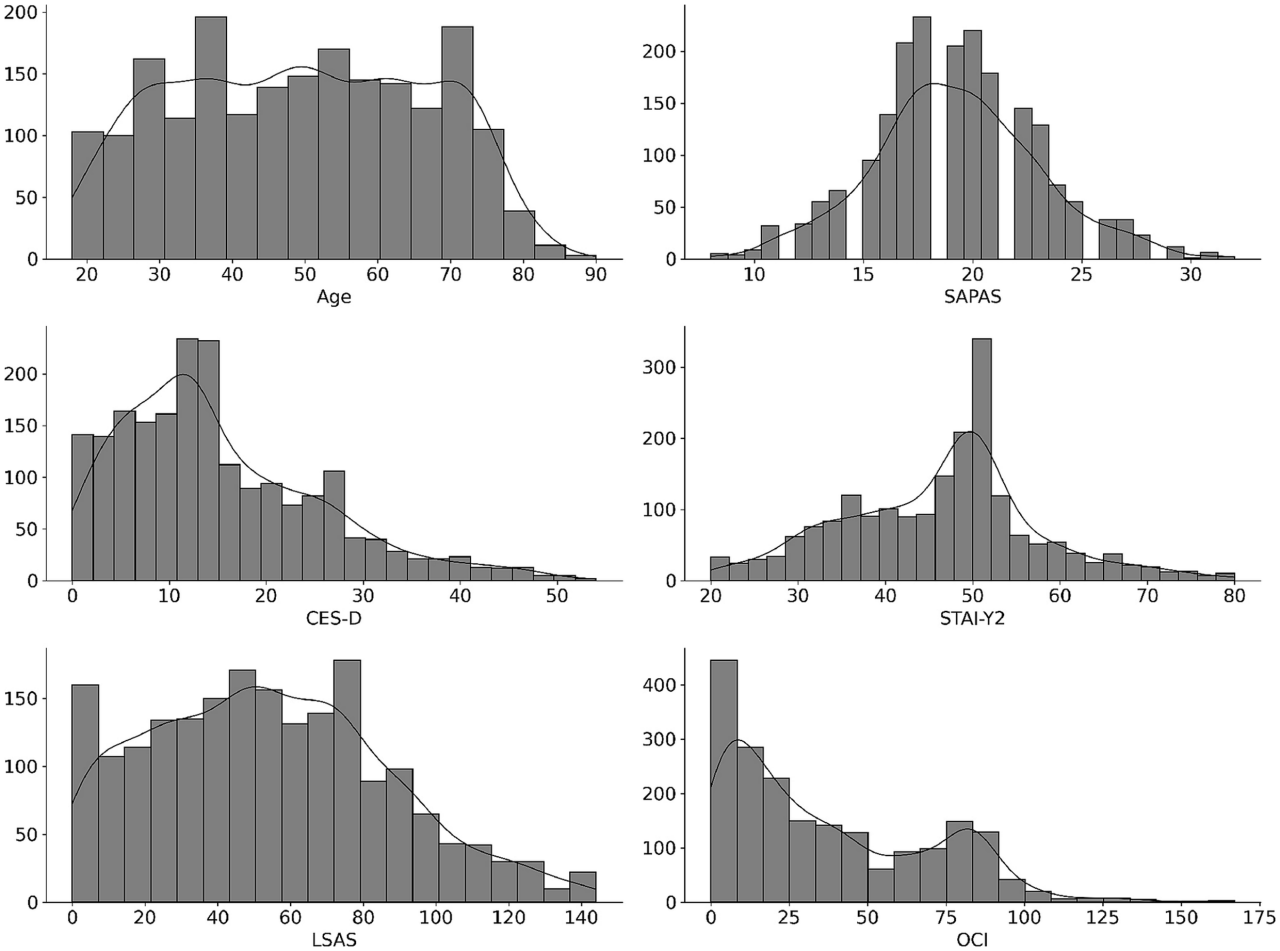
Distributions of scores in Study 2.*SAPAS*, the Japanese version of Standardised Assessment of Personality - Abbreviated Scale *CES-D*, Center for Epidemiologic Studies Depression Scale *STAI-Y2*, State–Trait Anxiety Inventory Form-Y2*LSAS*, Leibowitz Social Anxiety Scale *OCI*, Obsessive-Compulsive Inventory.

Descriptive values are provided in Table 7. The average score of SAPAS in Study 2 was 19.22 (SD = 3.84). There were no ceiling or floor effects. To support the convergent validity, we calculated correlations with SAPAS, and the results are shown in Table 8. Since scores were not normally distributed (Figure 3), we calculated Spearman’s *ρ*. All correlations were significant and moderate (CES-D: *ρ* = 0.50; STAI-Y2: *ρ* = 0.58; LSAS: *ρ* = 0.40; OCI: *ρ* = 0.40.)

**Table 7:** Descriptive statistics in Study 2.

	*M*	SD
SAPAS	19.22	3.84
CES-D	15.08	10.49
STAI-Y2	46.18	11.26
LSAS	53.92	32.98
OCI	37.40	32.25

SAPAS, the Japanese version of Standardised Assessment of Personality - Abbreviated Scale; CES-D, Center for Epidemiologic Studies Depression Scale; STAI-Y2, State–Trait Anxiety Inventory Form-Y2; LSAS, Leibowitz Social Anxiety Scale; OCI, Obsessive-Compulsive Inventory.

**Table 8:** Correlations with SAPAS-J in Study 2.

	*ρ_Spearman_*	*p*
CES-D	0.50	< 0.001
STAI-Y2	0.58	< 0.001
LSAS	0.40	< 0.001
OCI	0.40	< 0.001

CES-D, Center for Epidemiologic Studies Depression Scale; STAI-Y2, State–Trait Anxiety Inventory Form-Y2; LSAS, Leibowitz Social Anxiety Scale; OCI, Obsessive-Compulsive Inventory.

## 4 Discussion

In Study 1, we confirmed the factorial structure, reliability, and validity of SAPAS-J. We tested two factorial models. One was a single-factor model based on the assumption that SAPAS-J simply measures the transdiagnostic general PD traits. The other was a higher-order model reflecting the three-factor structure of SAPAS-J, corresponding to clusters described in DSM-5. This second model measures general PD traits as a common higher-order latent variable including them. Considering that SAPAS was developed based on the cluster model of DSM, we expected that this model would better explain SAPAS structure. While the single-factor model indicated acceptable *SRMR* and not quite sufficient *CFI* and *RMSEA*, all indices in the high-order model were good or acceptable. Hence, we consider the SAPAS-J to be a higher-order, single-factor scale containing three factors. Although this result did not suggest an alternative use of SAPAS-J, it supposes that SAPAS is a scale measuring the common feature of three clusters of PD, and helps future discussions about results using this questionnaire.

Although its internal consistency was not high, it was equal to or better than that of the original version (*α_Cronbach_* = 0.68; Moran et al., 2003). The original SAPAS intended to capture the broad character of personality disorder with the fewest possible items. The low correlations between items were reasonable. Because the test–retest reliability of SAPAS-J was very good, overall, the reliability of SAPAS-J was demonstrated.

With our two studies, we demonstrated its validity. We showed sufficiently strong correlations between PDQ-R and SAPAS-J. Almost half of the information of PDQ-R, the 121-item questionnaire, was explained by this 8-item questionnaire, confirming the effectiveness of SAPAS-J. SAPAS-J also indicated adequate correlations between depressive, anxious, and positive moods among undergraduates. Furthermore, by expanding the survey to the general population, we demonstrated correlations with more pathological symptoms than depressive and anxious moods in the preceding days. Moderate correlation with depressive symptoms, trait anxiety, and scales relating to two anxiety disorders, i.e., OCD and SAD, in people with a broad range of ages and characteristics was indicated. Though disorders tend to occur depending on the category (i.e., schizotypal, borderline, etc.) of PD, the validity of SAPAS-J as a screening tool for general PD traits is supported.

SAPAS-J demonstrated sufficient reliability, and the results support its convergent and structural validity. Considering that it is a translation of a widely used screening tool, its content validity is guaranteed. However, the current validation had some limitations. First, our samples were collected only from a non-clinical population, and further validation with clinical samples is needed. Nevertheless, we demonstrated that its reliability and factorial structure correspond to DSM-5 clusters in a non-clinical population. The present research supports proceeding to validate SAPAS-J with clinical samples. Future studies should validate SAPAS-J, with the Structured Clinical Interview for DSM (SCID; First et al., 2015) as a candidate reference standard, as the original (Moran et al., 2003) and other language versions (Germans et al., 2008; Merlhiot et al., 2014) used it. We used PDQ-R to validate SAPAS, as it produces adequate variance in scores among the general population (Trull et al., 1993). However, semi-structured interviews such as SCID or the International Personality Disorder Examination (Loranger, 1994) are required to demonstrate concurrent validity, since the false-positive rate of PDQ-R tends to be high (Hyler et al., 1992).

While validation for the clinical use of SAPAS-J is limited, sufficient validity was demonstrated for its use in the context of psychopathological analog research. As a recent trend in research and clinical understanding, the model of PD is shifting from the conventional categorical/cluster model to a dimensional model (American Psychiatric Association, 2013a). Since PD is understood as a continuum, the severity of which distributes dimensionally (Bagge and Trull, 2003; Brud and Cieciuch, 2022), the analog study, recruiting members of the general population and attempting to reveal psychopathological mechanisms of PD, is meaningful (Carr and Francis, 2010). While SAPAS was developed based on the cluster model, and we found that its structure includes three factors, we think it is compatible with the dimensional model. The three factors in SAPAS converged on the higher-order single factor, and this is considered to be the common feature of PD. Although further research is necessary to validate this hypothesis, we postulate that the common factor identified in our study is likely to be preserved even in the dimensional model. Therefore, we argue that SAPAS can be effectively utilized in future research adopting the dimensional approach to PDs. It would also be beneficial to clarify the relationship between elements of PD dimensions and SAPAS score. Correlation between SAPAS and some questionnaires measuring personality functioning, e.g., DSM-5 Levels of Personality Functioning Questionnaire; Huprich et al. (2018), and pathological personality traits, e.g., Personality Inventory for DSM-5; Krueger et al. (2012), would better clarify the concept that SAPAS assesses. Furthermore, considering that some previous research reported evidence suggesting substantial variation in psychopathology when the same mental disorder co-occurs with a personality disorder (Richman and Unoka, 2015; Chechko et al., 2016), assessing PD traits can also be worthwhile in analog studies seeking to investigate the pathology of psychological disorders other than PD. SAPAS-J requires so little time that researchers can assess PD traits with little burden on participants. Moreover, it can handle the heterogeneity raised by the comorbidity of PD in various mental disorder analog studies. Although there are other self-reporting questionnaires for PD traits besides SAPAS-J, its brevity is a significant advantage.

## Data availability statement

The datasets presented in this article are not readily available for the following reasons: The data from Study 1 cannot be shared publicly as consent for its dissemination was not obtained from the participants. The data used in Study 2 is subject to ongoing research; however, it is planned to be shared after the research concludes. Requests to access the datasets should be directed to the corresponding author.

## Ethics statement

The studies involving humans were approved by ATR Review Board Ethics Committee, Ethics Review Committee on Research with Human Subjects at the Waseda University. The studies were conducted in accordance with the local legislation and institutional requirements. The ethics committee/institutional review board waived the requirement of written informed consent for participation from the participants or the participants’ legal guardians/next of kin because the participants were instructed that their participation in the study would be considered as consenting to respond to the survey, and the study was conducted accordingly.

## Author contributions

IK: Conceptualization, Formal analysis, Methodology, Writing – original draft, Funding acquisition. TH: Conceptualization, Data curation, Formal analysis, Investigation, Writing – review & editing, Methodology. MN: Investigation, Writing – review & editing, Data curation. AY: Investigation, Writing – review & editing, Data curation. MH: Investigation, Writing – review & editing, Funding acquisition, Data curation. HK: Methodology, Project administration, Supervision, Writing – review & editing, Conceptualization. ST: Funding acquisition, Project administration, Supervision, Writing – review & editing, Investigation.
