# Supplementary material for: Psychometric properties of the Japanese version of the standardised assessment of personality abbreviated scale
Source: Front Psychol. 2024 Feb 6;14:1339902. doi: 10.3389/fpsyg.2023.1339902 (PMC10878311; doi:10.3389/fpsyg.2023.1339902)
Supplement: Supplementary file 1 [file Data_Sheet_1.docx]

# [教示文]

以下の項目内容を読んで、普段のあなた自身について、
あてはまると思うものに、各項目1箇所ずつ○をつけて下さい。

# [選択肢]

1. あてはまらない / 2. あまりあてはまらない / 3.ややあてはまる / 4. あてはまる

# [項目]

1. 基本的に、あなたは友人関係を築いたり、維持したりすることに難しさを感じますか？

2. あなたは普段、一人でいたり、一人で行動したりすることを好みますか？

3. 基本的に、あなたは他人を信頼できますか？*

4. あなたは普段、気が短いほうですか？

5. あなたは普段、衝動的な人ですか？

6. あなたは普段、心配性ですか？

7. 基本的に、あなたはよく誰かにひどく依存しますか？

8. 基本的に、あなたは完ぺき主義ですか？

* Item 3 is reversed item. Please use total scores when using SAPAS-J. For other up-to-date information on the use of SAPAS-J will be uploaded on <https://osf.io/sm6eh/>
